# Epidemiology of paediatric presentations with musculoskeletal problems in primary care

**DOI:** 10.1186/s12891-018-1952-7

**Published:** 2018-02-06

**Authors:** Albert Tan, Victoria Y. Strauss, Joanne Protheroe, Kate M. Dunn

**Affiliations:** 1 Arthritis Research UK Primary Care Centre, Research Institute for Primary Care & Health Sciences, Keele University, Keele, Staffordshire ST5 5BG UK; 2 Centre for Statistics in Medicine, Oxford Clinical Trial Research Unit , Nuffield Department of Orthopaedics, Rheumatology and Musculoskeletal Sciences, University of Oxford Botnar Research Centre, Windmill Road, Oxford, OX3 7LD UK

**Keywords:** Musculoskeletal, Paediatric, Primary care

## Abstract

**Background:**

Musculoskeletal disease is a common cause of morbidity, but there is a paucity of musculoskeletal research focusing on paediatric populations, particularly in primary care settings. In particular, there is limited information on population consultation frequency in paediatric populations, and frequency varies by age and sex. Few studies have examined paediatric musculoskeletal consultation frequency for different body regions. The objective was to determine the annual consultation prevalence of regional musculoskeletal problems in children in primary care.

**Methods:**

Musculoskeletal codes within the Read morbidity Code system were identified and grouped into body regions. Consultations for children aged three to seventeen in 2006 containing these codes were extracted from recorded consultations at twelve general practices contributing to a general practice consultation database (CiPCA). Annual consultation prevalence per 10,000 registered persons for the year 2006 was determined, stratified by age and sex, for problems in individual body regions.

**Results:**

Over 8 % (8.27%, 95% CI 7.86 to 8.68%) of the 16,862 children consulted with a musculoskeletal problem during 2006. Annual consultation prevalence for any musculoskeletal problem was significantly higher in males than females (male: female prevalence ratio 1.18, 95% CI 1.06 to 1.31). Annual consultation prevalence increased with age and the most common body regions consulted for were the foot, knee and back all of which had over 100 consultations (109, 104 and 101 respectively) per 10,000 persons per year.

**Conclusions:**

This study provides new and detailed information on patterns of paediatric musculoskeletal consultations in primary care. Musculoskeletal problems in children are varied and form a significant part of the paediatric primary care workload. The findings of this study may be used as a resource for planning future studies.

## Background

Musculoskeletal problems are a common reason for healthcare consultation, with an estimated 24% of the population seeking primary healthcare from a General Practitioner (GP) each year [1]. Around 7% of children visit primary care for musculoskeletal problems each year [1], and yet the majority of musculoskeletal research has focused on adult populations. There is a paucity of musculoskeletal research investigating the paediatric population [2].

Population-based and school-based studies have demonstrated that pain is a common feature of childhood [3] and that that the majority of pain in childhood has a musculoskeletal cause. [4, 5] In children, pain is the most common symptom of a musculoskeletal problem, although other symptoms include limping, stiffness, muscle weakness and fatigue [6]. A systematic review of population-based studies in children and adolescents estimated the prevalence of musculoskeletal pain to be between 8.5% and 40% (recall periods from one week to six months) with the knee, back and neck suggested as common sites of musculoskeletal pain [7].

There is limited information about consultations for paediatric musculoskeletal problems in primary care. Very few studies provide information broken down into more than two age-groups within the paediatric population (e.g. [8] and [6] use three age-groups), and given the rapid changes in musculoskeletal problems with age in general paediatric populations [9], this is a large gap. In addition, few studies provide comparable data for different body regions, which means comparisons of prevalence across different body regions is difficult, and there is limited international data (e.g. three of four studies identified in a search were Dutch [10–12] plus one from the UK [1].

The aim of this study was to describe the annual consultation prevalence of regional musculoskeletal problems for children aged three to 17 years in primary care. Particular objectives were to examine figures by age-group, sex and body region.

## Methods

### CiPCA

This was an analysis of all healthcare visits for musculoskeletal problems among children in a UK primary care medical record database. The study used the Consultations in Primary Care Archive (CiPCA), which is an ongoing primary care medical record database containing anonymised data on all consultations (contacts between patients and healthcare professionals) in 12 general practices in North Staffordshire, UK [13]. In the UK, general practice serves as the first point of contact for health care for over 95 % of the population.

All practices within CiPCA document consultations using the Read clinical classification system which provides Read codes and Read terms. This is a hierarchical coding system which contains between one and five characters and is commonly used in UK primary care. Each extra character represents more information regarding a consultation. For example, N is the chapter for musculoskeletal/connective tissue, “N07” is internal derangement of knee and “N071C” is the code for old tear of lateral meniscus. A healthcare professional may assign one or more Read codes to a consultation. Practices within CiPCA are required to code clinical consultations to a high standard and undergo an annual cycle of training and assessment in computerised morbidity coding. Data regarding consultations in the calendar year 2006 were examined and in this year, 97 % of GP consultations recorded in CiPCA had one or more morbidity codes assigned [14].

Ethical approval for CiPCA was granted by the North Staffordshire Research Ethics Committee. Separate ethical approval was not required for this study.

### Study population and musculoskeletal problems definitions

The registered population as of 1st July 2006 was 100,758 patients. A total of 16,862 registered patients were aged between three and 17 years.

To define musculoskeletal presentations for this study, we used the same Read codes that were used in a previously published study by Jordan et al. in which they determined the annual consultation prevalence of regional musculoskeletal problems in primary care [1]. In the study by Jordan et al., all Read codes potentially related to pain or musculoskeletal disorders were identified, a total of 5098. Consultations for children aged three to 17 years occurring during the calendar year 2006 and containing any of these 5908 musculoskeletal Read codes were identified.

The majority of these 5908 codes come from the musculoskeletal diagnosis chapter (N) and injury chapter (S) for example, knee joint pain (N0946) and knee sprain (S54), respectively. They also contain musculoskeletal pain symptoms under the symptoms chapters of R and 1, e.g. general aches and pains (R00z2) and aching muscles (1DCC). The full list can be accessed by www.keele.ac.uk/mrr. We followed the same approach used in the study by Jordan et al. [1] in which the 5908 Read codes were each assigned to 48 body regions. In that study, a framework for allocation of codes to body regions was created using a sample of 100 codes. Four GPs were trained in these use of this and assigned codes to body regions. The term of unspecified was used when a region could not be assigned (for example aching muscles).

In this study, primary care consultations were included if they occurred at the practice, via home visit or were telephone consultations; this excluded secondary care appointments or accident and emergency visits, as this study focused on primary care appointments.

### Statistical analysis

The study population was divided into four age-groups: pre-school (three to five years); school age (six to nine years); early adolescence (10 to 13 years); late adolescence (14 to 17 years). The division is based on that used previously [8], expanded to include 14 to 17 year olds.

Annual consultation prevalence was calculated for all musculoskeletal problems, for each body region, stratified by age-group and sex. Annual consultation prevalence was defined as the proportion of patients registered with contributing GP practices at mid-year (1st July) of 2006 who had at least one musculoskeletal consultation as defined above. Annual consultation prevalence for each body region was defined as the proportion of the patients who had at least one consultation in the year containing a Read code assigned to that body region.

To compensate for demographic differences between the CiPCA and the general population of England and Wales, standardised annual consultation prevalence figures were calculated and presented by weighting each age/sex-specific rate based on the proportion of age/sex group in the general population of England and Wales in 2006 [15]. Pearson’s chi squared test was used to test the statistical significance of differences between age and sex groups in prevalence rates of regional and overall musculoskeletal consultation. In addition, a standardised prevalence ratio of the standardised prevalence rate in males to that in female. This standardised ratio was calculated by Negative binomial regression that was deemed to account for small prevalence in large sample sizes [16]. All analyses were conducted using STATA v12.0.

## Results

**Fig. 1:**
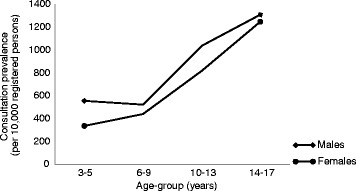
Annual consultation prevalence for all musculoskeletal problems per 10,000 registered persons (aged 3 to 17 years) by sex and age-group

Eight percent (8.27%; 95% CI 7.86 to 8.68%) of children consulted at least once with a musculoskeletal problem during 2006. The annual consultation prevalence for any musculoskeletal problem was significantly higher (*p* = 0.04) in males than females (male: female prevalence ratio 1.18, 95% CI 1.06 to 1.31). The annual consultation prevalence were similar in the two youngest age-groups, but then increased with age-group for both sexes (see Fig. 1).

### Consultation prevalence by body region

The most common body regions for paediatric musculoskeletal consultations were the foot, knee and back (see Table 1), all of which had over 100 consultations (109, 104 and 101 respectively) per 10,000 persons per year.

**Table 1:** Standardised annual consultation prevalence per 10,000 registered persons (aged 3 to 17 years) for the 12 most common regional problems

Body region	Rate per 10,000 persons (95% CI) ^a^		Male: female prevalence ratio (95% CI) ^a^	
Foot	109	(93 to 124)	0.97	(0.72 to 1.29)
Knee	104	(89 to 119)	1.42	(1.05 to 1.91)
Back (any ^b^)	101	(86 to 115)	1.00	(0.75 to 1.35)
Chest	81	(67 to 94)	1.38	(0.98 to 1.93)
Head	72	(59 to 85)	1.32	(0.92 to 1.90)
Hand	49	(38 to 62)	1.37	(0.88 to 2.11)
Neck	45	(35 to 56)	0.97	(0.63 to 1.52)
Ankle	37	(28 to 46)	1.07	(0.66 to 1.76)
Pelvis	36	(27 to 45)	0.89	(0.54 to 1.46)
Hip	26	(18 to 33)	0.74	(0.41 to 1.34)
Wrist	19	(13 to 26)	0.77	(0.39 to 1.52)
Shoulder	13	(7 to 18)	0.46	(0.19 to 1.12)

^a^ males and females; age sex-standardised based on population figures for England and Wales in 2006 [15]

^b^ includes consultations coded as upper back, lower back or back

Rates of consultation increased with increasing age-group for most body regions, with statistically significant age trends (*p* < 0.05) found in all regions described with the exception of the head and hip (see Tables 2 and 3). Back problems went from a relatively infrequent cause of musculoskeletal complaints in under nines to being the most common cause of musculoskeletal complains in the 14–17 year old age-group for both sexes (228 per 10,000 persons in males; 95% CI 178 to 293; 205 per 10,000 persons in females; 95% CI 157 to 268).

**Table 2:** Standardised annual consultation prevalence per 10,000 registered persons (aged 3 to 17 years) for each body region - males by age-group

Male (age-group in years) prevalence									
Region	Total ^a^	3–5	(95% CI)	6–9	(95% CI)	10–13	(95% CI)	14–17	(95% CI)
Ankle	37	13	(4 to 49)	32	(15 to 66)	53	(30 to 93)	46	(26 to 80)
Back	48	7	(1 to 38)	18	(7 to 47)	22	(9 to 52)	110	(77 to 158)
Back (any ^b^)	101	13	(4 to 49)	27	(13 to 59)	93	(61 to 142)	228	(178 to 293)
Chest	81	0	(0 to 26)	77	(48 to 123)	93	(61 to 142)	167	(125 to 224)
Elbow	8	0	(0 to 26)	9	(2 to 33)	9	(2 to 32)	23	(10 to 50)
Foot	109	67	(36 to 123)	45	(25 to 83)	182	(134 to 245)	118	(83 to 167)
Hand	49	33	(14 to 78)	36	(18 to 72)	58	(34 to 98)	88	(58 to 131)
Head	72	114	(71 to 181)	73	(45 to 118)	89	(57 to 136)	65	(32 to 89)
Head/neck	12	27	(10 to 69)	14	(5 to 40)	4	(1 to 25)	11	(4 to 34)
Hip	26	7	(1 to 38)	23	(0 to 17)	31	(15 to 64)	23	(10 to 50)
Knee	104	67	(36 to 123)	64	(38 to 107)	124	(86 to 179)	206	(158 to 267)
Limb	5	0	(0 to 26)	14	(5 to 40)	4	(1 to 25)	8	(2 to 28)
Lower back	49	7	(1 to 38)	5	(1 to 26)	66	(40 to 109)	110	(77 to 158)
Lower leg	28	7	(1 to 38)	0	(0 to 17)	66	(40 to 109)	65	(32 to 89)
Lower limb	52	94	(56 to 157)	50	(28 to 89)	35	(18 to 70)	72	(46 to 113)
Neck	45	20	(7 to 59)	32	(15 to 66)	58	(34 to 98)	61	(38 to 99)
Pelvis	36	0	(0 to 26)	32	(15 to 66)	40	(21 to 76)	53	(32 to 89)
Shoulder	13	0	(0 to 26)	5	(1 to 26)	13	(5 to 39)	11	(4 to 34)
Thigh	6	0	(0 to 26)	0	(0 to 17)	18	(7 to 45)	19	(8 to 44)
Upper limb	15	13	(4 to 49)	0	(0 to 17)	18	(7 to 45)	23	(10 to 50)
Wrist	19	0	(0 to 26)	5	(1 to 26)	13	(5 to 39)	42	(23 to 75)
All regions	827	556	(450 to 684)	523	(437 to 624)	1037	(918 to 1169)	1309	(1186 to 1444)

^a^males and females; age-sex standardised based on population figures for England and Wales in 2006

^b^includes consultations coded as upper back, lower back or back

**Table 3:** Standardised annual consultation prevalence per 10,000 registered persons (aged 3 to 17 years) for each body region - females by age-group

Female (age-group in years) prevalence									
Region	Total ^a^	3–5	(95% CI)	6–9	(95% CI)	10–13	(95% CI)	14–17	(95% CI)
Ankle	37	7	(1 to 40)	15	(5 to 43)	45	(24 to 82)	62	(38 to 101)
Back	48	0	(0 to 27)	29	(13 to 63)	54	(31 to 93)	105	(72 to 152)
Back (any ^b^)	101	0	(0 to 27)	44	(23 to 83)	112	(76 to 164)	205	(157 to 268)
Chest	81	0	(0 to 27)	63	(37 to 107)	49	(27 to 88)	132	(94 to 184)
Elbow	8	0	(0 to 27)	5	(1 to 27)	4	(1 to 25)	4	(1 to 22)
Foot	109	57	(29 to 113)	58	(33 to 101)	170	(124 to 232)	136	(98 to 188)
Hand	49	29	(11 to 73)	5	(1 to 27)	58	(34 to 99)	66	(41 to 105)
Head	72	72	(39 to 131)	58	(33 to 101)	31	(15 to 64)	85	(56 to 129)
Head/neck	12	7	(1 to 40)	10	(3 to 35)	9	(2 to 33)	16	(6 to 40)
Hip	26	7	(1 to 40)	29	(13 to 63)	27	(12 to 58)	47	(27 to 81)
Knee	104	29	(11 to 73)	19	(8 to 50)	94	(61 to 143)	171	(127 to 228)
Limb	5	14	(4 to 52)	5	(1 to 27)	0	(0 to 17)	0	(0 to 15)
Lower back	49	0	(0 to 27)	0	(0 to 19)	54	(31 to 93)	105	(72 to 152)
Lower leg	28	0	(0 to 27)	5	(1 to 27)	40	(21 to 76)	19	(8 to 45)
Lower limb	52	43	(20 to 93)	39	(20 to 76)	22	(10 to 52)	66	(41 to 105)
Neck	45	21	(7 to 63)	24	(10 to 56)	54	(31 to 93)	74	(47 to 115)
Pelvis	36	14	(4 to 52)	5	(1 to 27)	31	(15 to 64)	89	(59 to 133)
Shoulder	13	14	(4 to 52)	0	(0 to 19)	18	(7 to 46)	35	(18 to 66)
Thigh	6	0	(0 to 27)	0	(0 to 19)	0	(0 to 17)	4	(1 to 22)
Upper limb	15	7	(1 to 40)	15	(5 to 43)	13	(5 to 39)	27	(13 to 56)
Wrist	19	7	(1 to 40)	10	(3 to 35)	22	(10 to 52)	43	(24 to 76)
All regions	827	336	(254 to 444)	440	(360 to 537)	818	(711 to 939)	1248	(1126 to 1381)

^a^males and females; age-sex standardised based on population figures for England and Wales in 2006

^b^includes consultations coded as upper back, lower back or back

For both sexes consultations with a foot complaint increased sharply from the 6–9 age-group to the 10–13 age-group. The foot was the most common region of musculoskeletal problems in the 10–13 age group for both males (182 per 10,000 persons per year; 95% CI 134 to 245) and females (170 per 10,000 persons per year; 95% CI 124 to 232) before declining in the 14–17 year old age-group for both sexes.

Although a sex predisposition was suggested by the data for several body regions, the only body region for which a statistically significant sex trend was found for overall rates of consultation was the knee, which was more common in males (Rate ratio of males/females: 1.42 (95% CI: 1.05, 1.91)).

## Discussion

This paper presents new data on the population-based annual primary care consultation prevalence of paediatric musculoskeletal problems, including figures stratified by age-group, sex and body region. In total, over 8% of the paediatric population consulted at least once with a musculoskeletal problem during the study year, and there was a clear rise in prevalence by age-group. Consultations were slightly (but significantly) more common among males than females. The most common body regions for paediatric musculoskeletal consultations were the foot, knee and back, each with around 1% of the sample population seeking healthcare.

The broad age-specific figures presented are similar to previous UK estimates of musculoskeletal annual consultation prevalence [1, 17, 18], and support the limited evidence on increasing musculoskeletal consultation rates with age [18–21]. However, our findings provide more detailed information on this trend through the use of four age-groups. This trend for increasing numbers of consultations with age parallels the reported increases in population prevalence of musculoskeletal problems by age in childhood [7, 9].

The overall sex trend in this study was a higher rate of primary care musculoskeletal consultations among boys than girls (male: female prevalence ratio 1.18 (95%CI 1.06 to 1.31). This differs from primary care figures from adults, which indicated that consultations were more common in females [1].

Sex trends for musculoskeletal consultations are conflicting in previous studies. Jordan et al. [1] demonstrated a higher rate of musculoskeletal consultation in males (75.6 per 1000 per year in boys aged zero to 14 compared to 59.7 per 1000 per year in girls). De Inocencio [6] demonstrated a higher rate of musculoskeletal consultation in females (187 per 1000 per year in girls compared to 148 per 1000 per year in boys). McCormick et al. (1995) demonstrated a higher rate of musculoskeletal consultation in boys in the zero to five year age group. The pattern was reversed in the six to 15 year age group with girls consulting more frequently.

If the higher rate of consultations among boys is confirmed, further research may investigate whether this is due to higher propensity to consult among boys, perhaps driven by different parental concerns, or related to the type of musculoskeletal problems experienced or reported differing among boys and girls. Higher rates of musculoskeletal consultation in boys may be due higher rates of exercise or trauma-related musculoskeletal problems for which parents may be more inclined to bring children to consult for as compared to other musculoskeletal problems. The database used in this study was large with high-quality coding of consultations, and therefore facilitated more in-depth investigation of paediatric patterns by age, sex and body region than many previous studies. However, the database is based within a single region of the UK, which could limit its representativeness. The database has been shown to produce annual consultation prevalence rates for musculoskeletal problems which are comparable to a larger national general practice consultation database [13]. The database has also been used to make international comparisons of consultation prevalence figures, while taking differences in healthcare and recording systems into account [17]. In order to minimise any implications of using a local database, age and sex standardised figures have been presented here. Another potential limitation is that the data analysed was from a single year. There is not enough published data on musculoskeletal consultations to understand the implications of this, but it is possible that changes in both the prevalence and patterns of consultations may change over time.

## Conclusions

This study provides new information about primary care patterns of musculoskeletal consultations among children and young people in primary care. The findings reported here improve our understanding of paediatric musculoskeletal problems, provide data on the varied regional paediatric musculoskeletal workload in primary care, and will be useful for planning of healthcare and future research studies.

## Abbreviations

CI: Confidence interval
CiPCA: Consultations in primary care archive
GP: General practitioner
STATA v12.0: A statistical software package created by StataCorp; version 12
UK: United Kingdom
